# Identification and validation of RNA-binding protein SLC3A2 regulates melanocyte ferroptosis in vitiligo by integrated analysis of single-cell and bulk RNA-sequencing

**DOI:** 10.1186/s12864-024-10147-y

**Published:** 2024-03-04

**Authors:** Jingzhan Zhang, Fang Xiang, Yuan Ding, Wen Hu, Hongjuan Wang, Xiangyue Zhang, Zixian Lei, Tingting Li, Peng Wang, Xiaojing Kang

**Affiliations:** 1 Department of Dermatology and Venereology, People’s Hospital of Xinjiang Uygur Autonomous Region, Ürümqi, China; 2 Xinjiang Clinical Research Center for Dermatology and Venereology, Xinjiang, China; 3 Xinjiang Key Laboratory of Dermatology Research, Xinjiang, China

**Keywords:** Vitiligo, Single-cell sequencing, Bulk RNA sequencing, RNA-binding protein, SLC3A2, Ferroptosis

## Abstract

**Background:**

The pathogenesis of vitiligo remains unclear. The genes encoding vitiligo-related RNA-binding proteins (RBPs) and their underlying pathogenic mechanism have not been determined.

**Results:**

Single-cell transcriptome sequencing (scRNA-seq) data from the CNCB database was obtained to identify distinct cell types and subpopulations and the relative proportion changes in vitiligo and healthy samples. We identified 14 different cell types and 28 cell subpopulations. The proportion of each cell subpopulation significantly differed between the patients with vitiligo and healthy groups. Using RBP genes for unsupervised clustering, we obtained the specific RBP genes of different cell types in vitiligo and healthy groups. The RBP gene expression was highly heterogeneous; there were significant differences in some cell types, such as keratinocytes, Langerhans, and melanocytes, while there were no significant differences in other cells, such as T cells and fibroblasts, in the two groups. The melanocyte-specific RBP genes were enriched in the apoptosis and immune-related pathways in the patients with vitiligo. Combined with the bulk RNA-seq data of melanocytes, key RBP genes related to melanocytes were identified, including eight upregulated RBP genes (*CDKN2A, HLA-A, RPL12, RPL29, RPL31, RPS19, RPS21*, and *RPS28*) and one downregulated RBP gene (*SLC3A2*). Cell experiments were conducted to explore the role of the key RBP gene *SLC3A2* in vitiligo. Cell experiments confirmed that melanocyte proliferation decreased, whereas apoptosis increased, after *SLC3A2* knockdown. *SLC3A2* knockdown in melanocytes also decreased the SOD activity and melanin content; increased the Fe^2+^, ROS, and MDA content; significantly increased the expression levels of TYR and COX2; and decreased the expression levels of glutathione and GPX4.

**Conclusion:**

We identified the RBP genes of different cell subsets in patients with vitiligo and confirmed that downregulating *SLC3A2* can promote ferroptosis in melanocytes. These findings provide new insights into the pathogenesis of vitiligo.

**Supplementary Information:**

The online version contains supplementary material available at 10.1186/s12864-024-10147-y.

## Background

Vitiligo is a chronic autoimmune skin disease with a global incidence of 0.5–2.0% in which the number of epidermal melanocytes decreases or disappears. The disease manifests as depigmented spots on skin and mucous membranes of different sizes and shapes. Vitiligo can significantly impact the quality of life of patients and can cause severe psychological disorders [1]. The pathogenesis of vitiligo has not yet been fully elucidated and involves multiple factors such as oxidative stress, autoimmunity, and genetic susceptibility [2, 3]. Melanocytes and T cells play key roles in the pathogenesis of vitiligo [4, 5]. However, continuous crosstalk between various cells in the dermis and epidermis of patients with vitiligo may seriously affect its development [6]. Various cells, including keratinocytes, fibroblasts, and CD8 + T cells, are involved in the pathogenesis of vitiligo. Studying the role of specific cells in the pathogenesis of vitiligo may be a breakthrough in further understanding the disease and developing targeted drugs.

Single-cell transcriptome sequencing (scRNA-seq) provides a technical means to reveal gene expression status at the single-cell resolution and reflects cell heterogeneity [7]. This technique has been used to study cell-specific gene expression profiles during the occurrence and development of vitiligo [8–10], and the important role of keratinocytes, dermal fibroblasts, and Treg cells in the pathogenesis of the disease have been elucidated. In scRNA-seq research, some scholars have detected the interaction between RNA-binding proteins (RBPs) and RNAs at the single-cell level [11].

RBPs are key effectors of gene expression [12]. Cell-specific RBPs regulate the expression of various cell-specific functional proteins, such as mRNA transcription and translation. Abnormal RBP expression is the basis of many diseases, including vitiligo [13–15]. For example, HMGB1 deficiency reduces oxidative damage via the Nrf2 pathway in human melanocytes [16]. However, the expression patterns of RBP genes in different skin cells and abnormal regulation related to vitiligo have not been reported. We speculate that RBP genes have specific expression characteristics in different skin cell types and that their abnormal regulation may be closely related to the occurrence and development of vitiligo. In our study, we performed comprehensive bioinformatic analysis, including scRNA-seq and bulk RNA-seq data, RBP genes, and cell experiments. We identified the RBP genes in different cell subsets in vitiligo for the first time. We identified the key RBP genes related to melanocytes in patients with vitiligo and confirmed that the RBP gene *SLC3A2* promotes ferroptosis in melanocytes. These results will provide theoretical support for studying the role of RBPs in the pathogenesis of vitiligo and new insights for studying melanocyte ferroptosis.

## Results

### scRNA-seq analysis of skin from patients with vitiligo and healthy donors identified distinct cell types

**Fig. 1:**
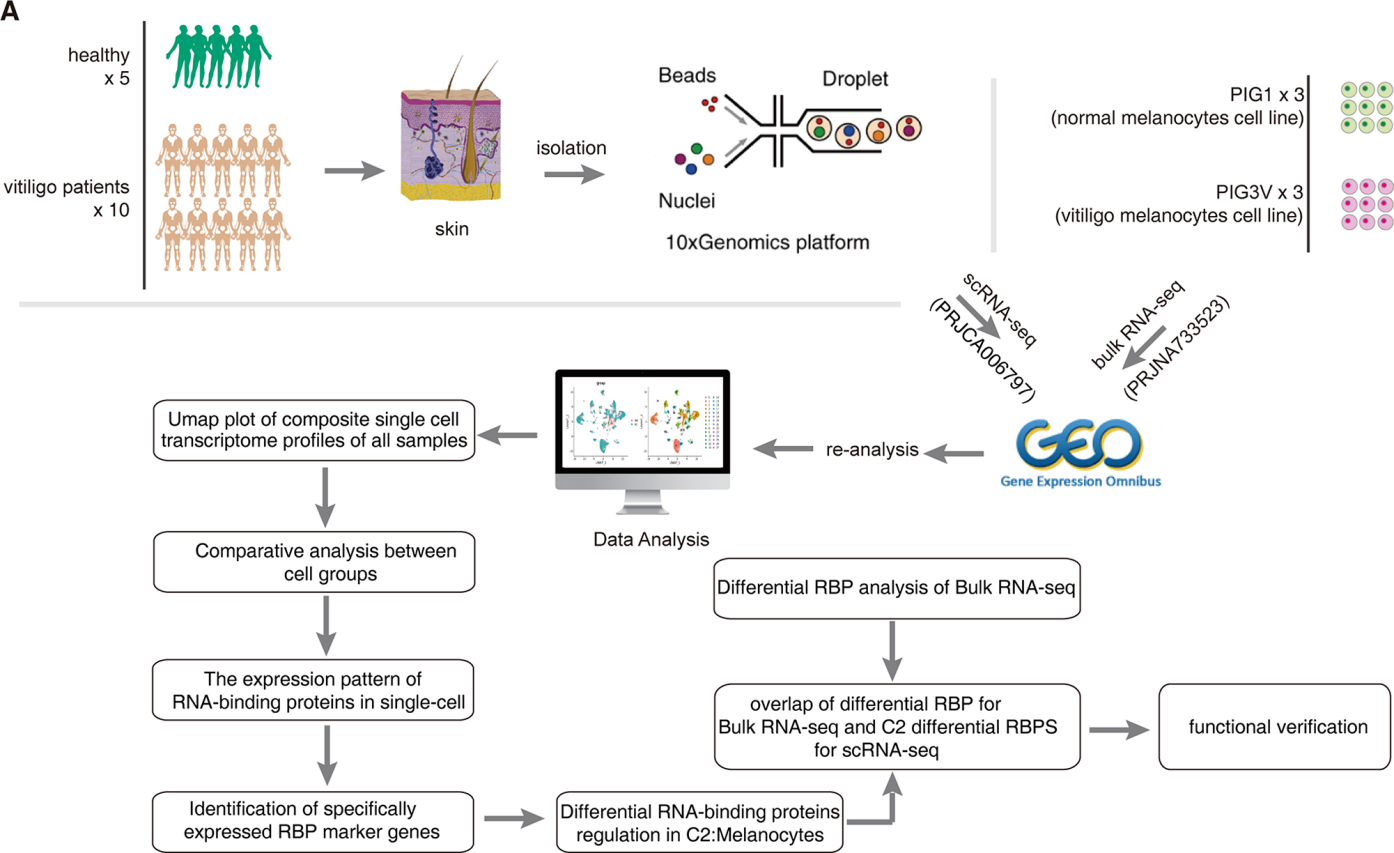
Schematic of sample preparation, scRNA-seq data processing, and data analysis

**Fig. 2:**
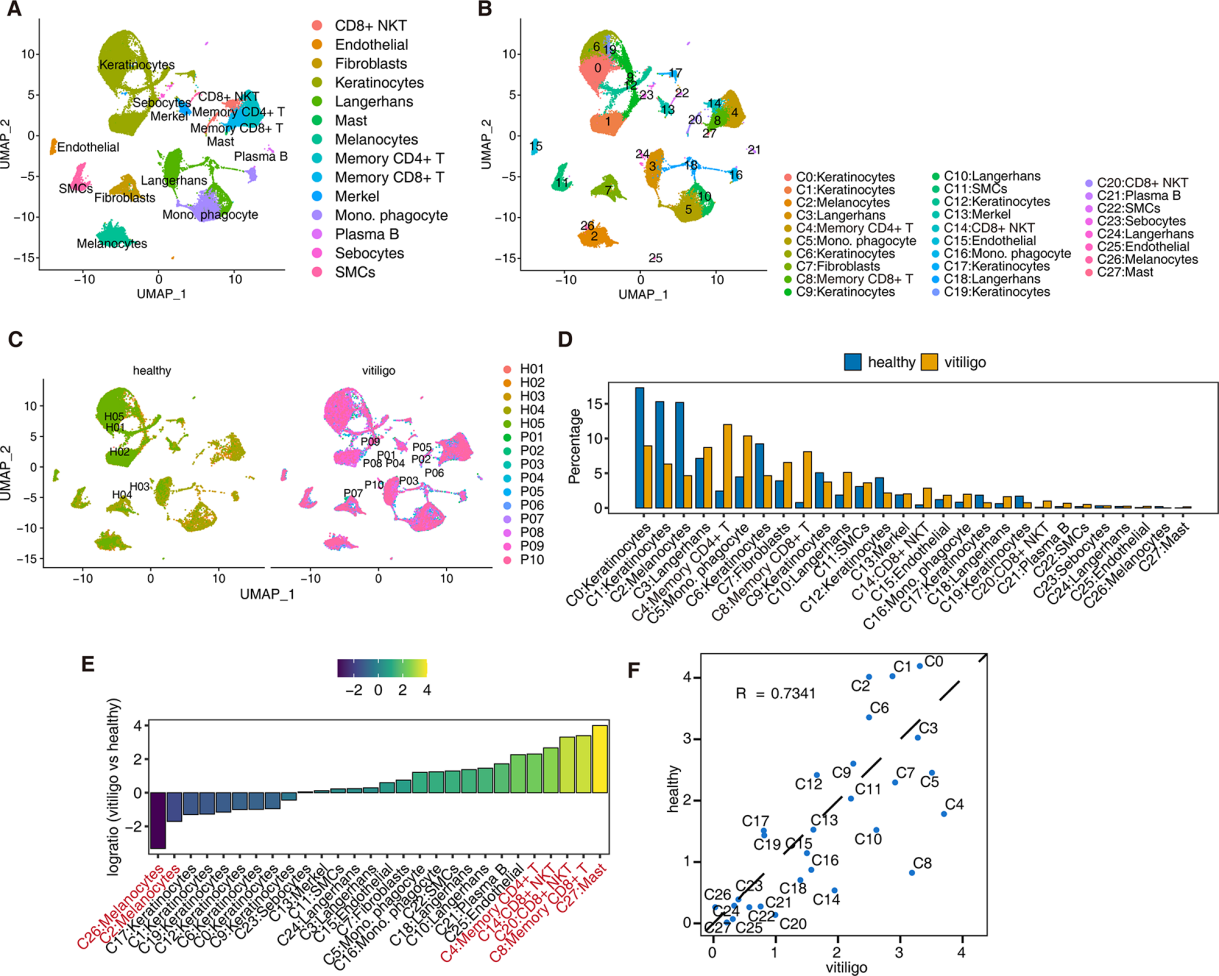
ScRNA-seq analysis of skin from healthy donors and patients with vitiligo identified distinct cell types. (**A**, **B**) UMAP plot of composite single-cell transcriptomic profiles from all 15 skin samples from healthy donors and patients with vitiligo. Colors indicate cell clusters along with annotations. Mono. Phagocyte: mononuclear phagocyte. (**C**) UMAP plot split by different sample groups. (**D**) Bar plot comparing the proportions of cell populations of each cell type within each sample group. (**E**) Rank order based on decreasing values of the relative frequency ratio between two sample groups. (**F**) Difference in the proportion of cell clusters between two sample groups

We collected scRNA-seq data from the skin samples of 15 individuals, of which 10 suffered from vitiligo, and 5 were healthy donors [10]. In addition, we obtained bulk RNA-seq data from three groups of vitiligo melanoma cell lines (PIG3V) and three groups of normal melanoma cell lines (PIG1) in the Sequence Read Archive (SRA) database for subsequent verification of the differentially expressed RBPs of melanocytes (Fig. 1). The scRNA-seq data from the 15 skin samples were subjected to strict quality control to obtain 50,339 single-cell expression profiles (Additional file 1). After normalization, a principal component (PC)-dimensionality-reduction analysis was performed, and the first 50 PCs were selected for UMAP dimensionality reduction and visualization (Additional file 2). Unbiased cluster analysis identified 28 cell subpopulations (clusters). Using ScType software combined with measuring the levels of cell markers reported in vitiligo skin, we identified 14 different cell types (Fig. 2A, B; Additional file 3). The cell composition between the patients with vitiligo and healthy groups differed; in particular, the immune T-cell groups were increased in the patients with vitiligo, whereas the melanocyte group was reduced (Fig. 2C).

We then analyzed the proportions of each cell subtype in each sample group. In the healthy group, melanocytes and keratinocytes accounted for most cells. In the patients with vitiligo, the proportion of these cells decreased, whereas that of T cells increased (Fig. 2D). Furthermore, we compared the relative proportion changes in each cell subpopulation between the patients with vitiligo and healthy group; compared with those in the healthy group, the proportions of C27 (mast cells), C8 (memory CD8 + T cells), C14/C20 (CD8 + NKT cells), and C4 (memory CD4 + T cells) cell subgroups increased in the patients with vitiligo, whereas C26/C12 (melanocytes) and almost all keratinocytes were reduced (Fig. 2E). There were significant differences in the proportion of cell clusters between the patients with vitiligo and healthy group (Fig. 2F). These results provide a panorama of cell composition and apparent changes in the skin of patients with vitiligo and healthy human skin, indicating that these cell populations may also differ in function (Additional file 4).

### Single-cell analysis revealed heterogeneity of cellular-specific RBPs in patients with vitiligo

**Fig. 3:**
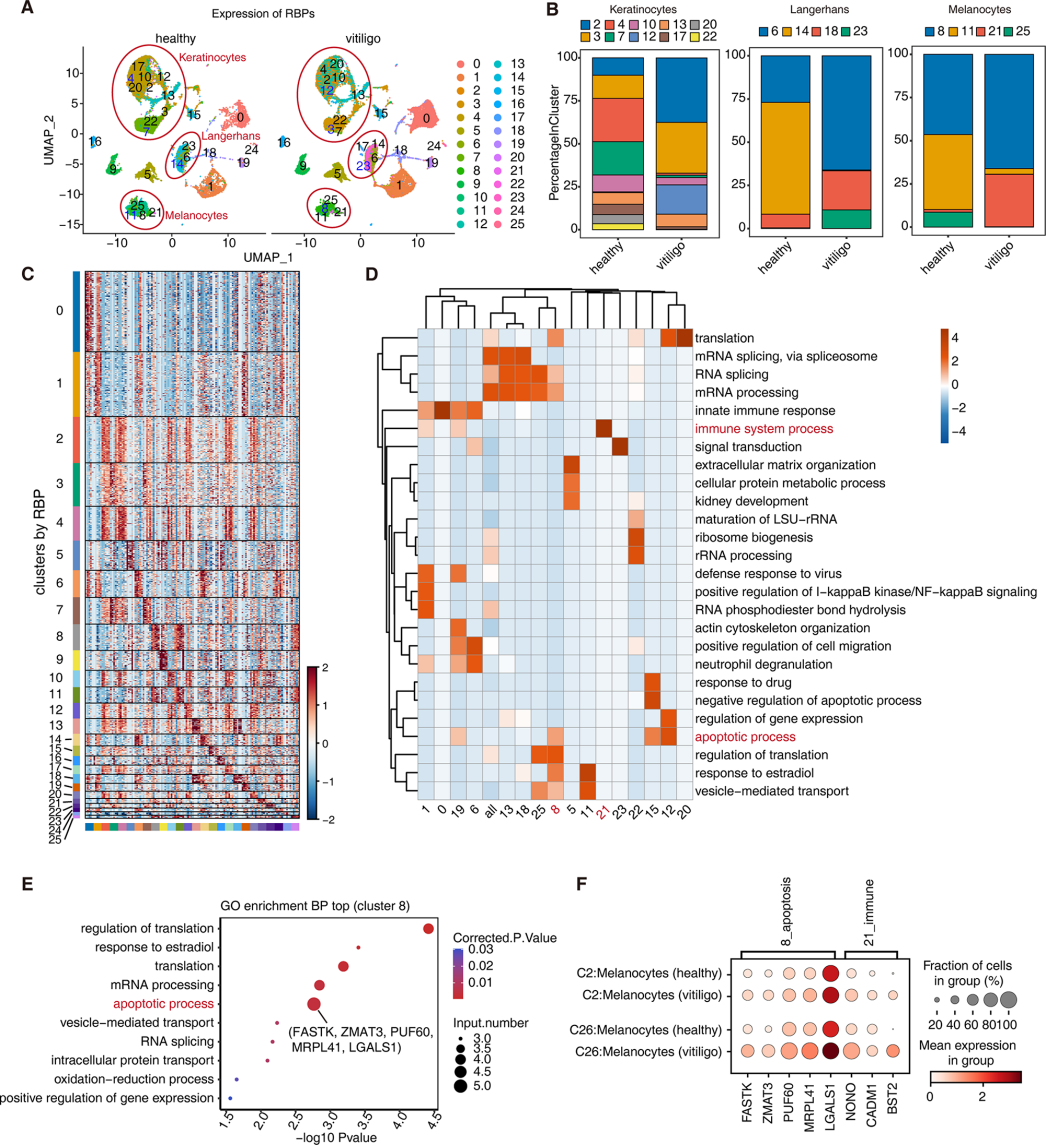
Single-cell analysis revealed heterogeneity and regulatory module of cell-specific RBPs in healthy donors and patients with vitiligo. (**A**) UMAP plot of scRNA-seq profile. Cells are colored according to cell clusters based on RBP-expression module. (**B**) Stacked bar plot showing relative proportions of cell populations based on RBP-expression module in keratinocytes, Langerhans, and melanocytes. (**C**) Unsupervised clustering heatmap showing relative expression (z score, row scaled) levels of top 5 representative markers in each cell population based on RBP-expression model. (**D**) Gene Ontology enrichment analysis of biological processes of top 50 marker genes of each cell type. Top three terms were selected for each cluster, and heatmap shows enrichment q-value of these terms (scaled by column). (**E**) Gene Ontology biological process of top 50 marker genes in Fig. 2D cluster8 based on RBP-expression module. (**F**) Dot plot showing expression of eight RBPs involved in apoptotic and immune system process terms from D in melanocytes. Dot color represents mean expression in each cluster; dot size indicates the percentage of cells expressing marker genes in each cluster

To explore the RBP-expression pattern in different cell populations, unsupervised clustering was performed using Seurat based on 2141 reported RBP genes. The RBP-expressing cell populations were highly cell type-specific; the proportions of the RBP-expressing cell populations of the same type in patients with vitiligo and healthy cells changed significantly, especially those of keratinocytes, Langerhans cells, and melanocytes (Fig. 3A, B; Additional file 5). For the keratinocytes, cluster4 and cluster7 RBP-expressing cell populations were dominant in the healthy samples, while cluster2, cluster3, and cluster12 RBP-expressing cell populations were dominant in samples from patients with vitiligo. In Langerhans cells, the cluster14 RBP-expressing cell populations were dominant in the healthy samples; in samples from patients with vitiligo, the cluster6 populations were dominant, and the cluster18 and cluster23 RBP-expressing cells increased in number. In the healthy samples, the cluster11 RBP-expressing melanocyte populations comprised a large proportion. In the samples from patients with vitiligo, the proportions of cluster11 and cluster25 were reduced, and those of cluster8 and cluster21 were increased (Fig. 3B). These results indicate that each cell type has subtypes with different RBP-expression patterns, and the relative proportions of these subtypes, which may represent different transcriptional patterns, correlated with disease states.

Distinct RBP-expressing cell populations showed particular RBP expression (Fig. 3C). These RBP-enriched functions exhibited common or specific features. For example, in the patients with vitiligo, the specifically increased melanocyte RBP-expression clusters were cluster8 and cluster21, and the marker RBPs of the RBP-expression clusters were enriched in the apoptosis and immune-related pathways, respectively (Fig. 3D). Cluster8, with more specific functional pathways, showed five RBP genes (*FASTK*, *ZMAT3*, *PUF60*, *MRPL41*, and *LGALS1*) in the apoptosis pathway (Fig. 3E), and three RBP genes in the immune function pathway were enriched in cluster21 (*NONO*, *CADM1*, and *BST2*). We observed differential expression of these eight RBPs in the two melanocyte subtypes (C26/C2) in the patients with vitiligo and healthy group. The proportion of cells expressing these RBPs increased significantly in the patients with vitiligo (Fig. 3F). Cluster12 keratinocyte RBP-expression groups, namely, *ZC3H12A, S100A9, CYCS*, and *EIF5A*, were also enriched in the apoptotic pathway in the patients with vitiligo. This suggests that the reduced number of keratinocytes and melanocytes in patients with vitiligo is closely related to the apoptotic pathway.

### Functional RBPs are largely regulated in melanocytes between patients with vitiligo and healthy individuals

**Fig. 4:**
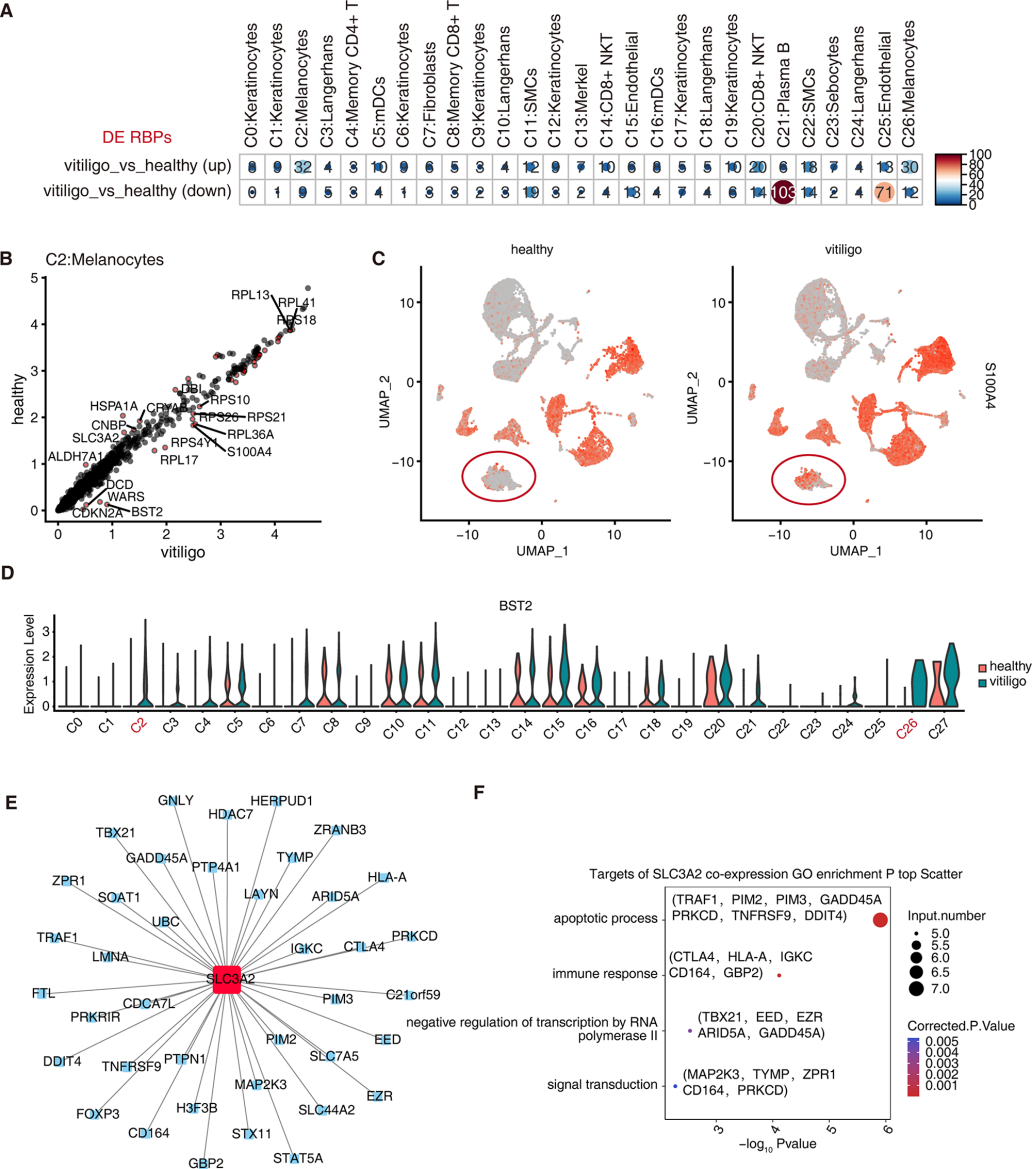
Functional RBPs are differentially regulated in melanocytes of patients with vitiligo and healthy donors. (**A**) Dot plot showing several differentially expressed RBPs in each cell type between vitiligo and healthy sample groups. (**B**) Scatter plot displaying differentially expressed RBPs in C2 melanocytes. Red color indicates significantly altered RBPs. (**C**) Gene expression levels of S100A4 in different sample groups represented in UMAP plot. (**D**) Violin plot of *BST2* in each cell type in different sample groups. (**E**) Cytoscape shows co-expression networks comprising SLC3A2 from C2 melanocyte cell clusters. Edges connect RBP-target gene pairs, while nodes represent genes. RBPs are displayed in larger font and red color. (**F**) Enriched biological processes of target genes for *SLC3A2* from E are shown

We analyzed and identified the RBP genes whose expression was significantly different in each cell subpopulation between the patients with vitiligo and healthy group using scRNA-seq data. More RBP genes were differentially expressed in melanocytes, CD8 + NKT cells, endothelial cells, B cells, and smooth muscle cells (Fig. 4A; Additional file 6). Some of these RBP genes were differentially expressed in multiple cell types, including *DCD*, *RPS26*, *RPS4Y1*, *HLA-A*, and *S100A4* (upregulated) and *HSPA1A*, *HSP90AA1*, *JUN*, *LGALS3*, and *HSPD1* (downregulated). We focused on the RBP genes that differed among the melanocyte populations (Fig. 4B). Among the upregulated RBP genes, some were related to the cell cycle or immune inflammation, such as *S100A4* (Fig. 4C) and *BST2* (Fig. 4D).

To confirm the potential regulatory function of *SLC3A2* in vitiligo C2 melanocytes, *SLC3A2* co-expression analysis was performed. The pySCENIC grn tool was used to predict the genes co-expressed with these RBPs; the RBP-target gene pairs with importance > 5 were screened according to the final importance value, and Cytoscape was used to draw the co-expression network between *SLC3A2* and its target genes (Fig. 4E). Among them, *TRAF1, PIM2, PIM3, DDIT4, CTLA4*, and *HLA-A* were significantly enriched in apoptosis and immune response pathways. *CD1641* was enriched in signal transduction pathways (Fig. 4F). These results confirmed that melanocyte-related *SLC3A2* is involved in the pathogenesis of vitiligo at the single-cell level.

### Bulk RNA-seq data validation of scRNA-seq results

**Fig. 5:**
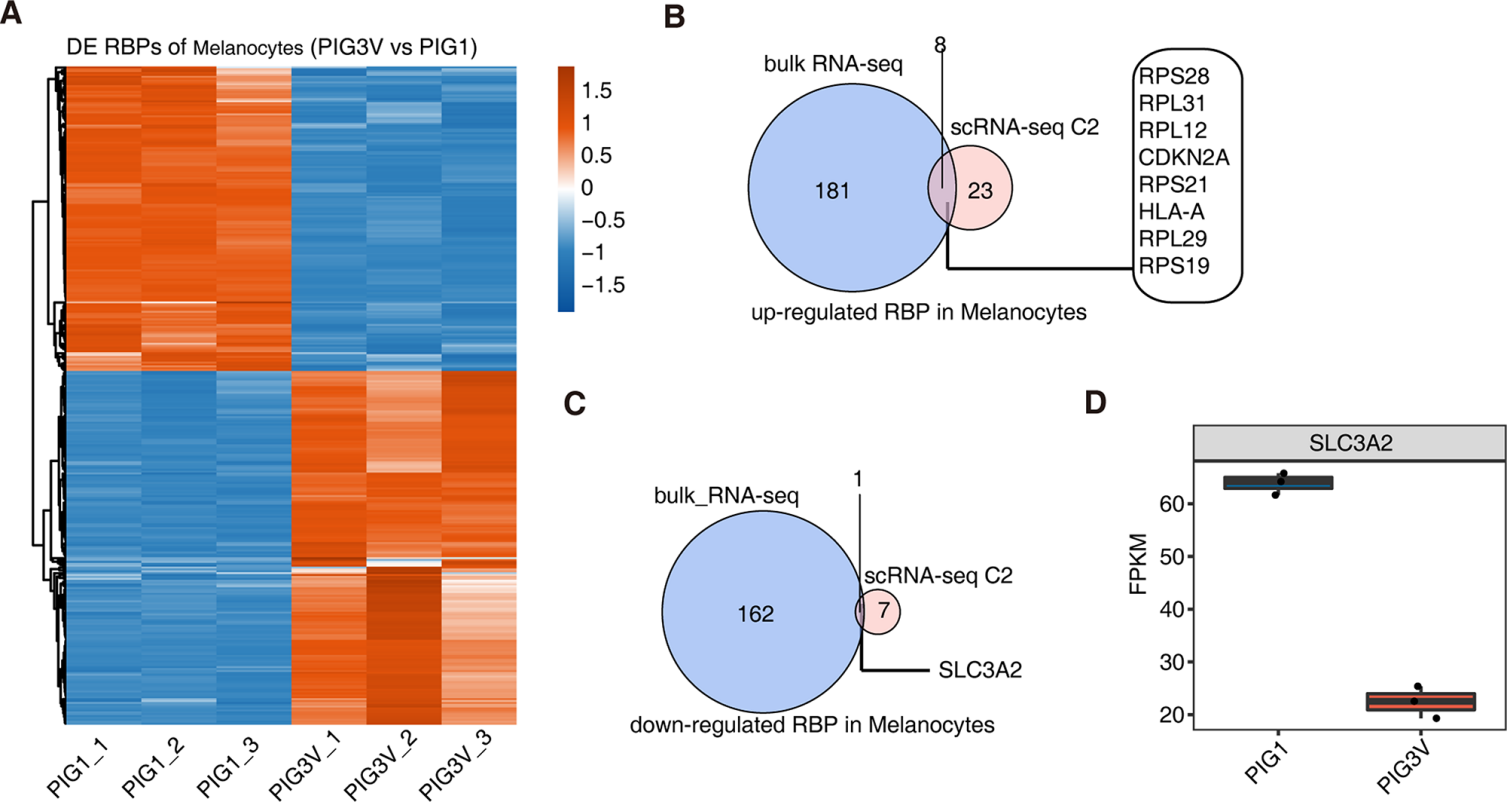
Integration of bulk RNA-seq data reveals significant differentially expressed RBPs in vitiligo melanocytes. (**A**) Expression heatmap of all 352 significant differentially expressed RBPs between PIG3V (vitiligo) and PIG1 (normal) melanocyte cell lines. (**B**) Venn diagram showing co-upregulated RBPs in bulk RNA-seq and C2 melanocyte cell clusters based on ScRNA-seq. (**C**) Venn diagram showing co-downregulated RBPs in bulk RNA-seq and C2 melanocyte cell clusters based on ScRNA-seq. (**D**) Box plot showing expression level of *SLC3A2*

To verify the expression of RBPs in melanocytes further, we obtained bulk RNA-seq data from normal (PIG1) and vitiligo (PIG3V) melanocytes [17]. Following quality control and differentially expressed gene (DEG) analysis, 3242 upregulated DEGs and 3209 downregulated DEGs were identified. The DEGs in the two cell lines were analyzed. Several RBP genes were differentially expressed (Fig. 5A; Additional file 7). The differential RBPs obtained through bulk RNA-seq showed limited intersection with the melanocyte-specific marker RBPs with increased or decreased expression in the patients with vitiligo. We screened nine melanocyte-associated co-differential RBPs from bulk RNA-seq and scRNA-seq data. These RBPs were differentially expressed at the tissue and cell levels and have high research significance in the pathogenesis of vitiligo. Eight of these RBP genes were upregulated—*CDKN2A*, *HLA-A*, *RPL12*, *RPL29*, *RPL31*, *RPS19*, *RPS21*, and *RPS28* (Fig. 5B). Among these, *HLA-A* is associated with immune inflammation [18]. *SLC3A2* was the only downregulated RBP gene, as verified via the bulk RNA-seq data (Fig. 5C, D). SLC3A2 is a putative spermidine transporter in melanocytes. *SLC3A2* downregulation promotes chondrocyte ferroptosis in osteoarthritis [19]. Whether this gene is involved in melanocyte ferroptosis has not yet been reported; therefore, we chose this gene for further studies.

### *SLC3A2* interference promotes ferroptosis of melanocytes

**Fig. 6:**
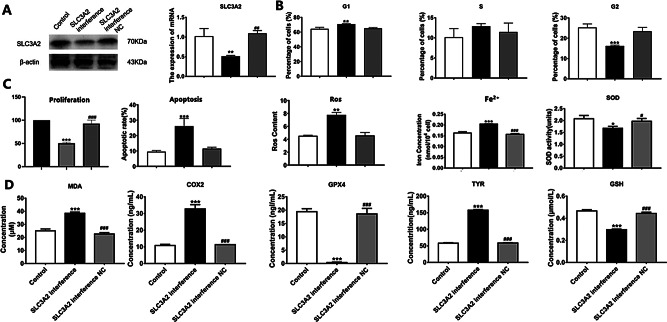
Downregulation of *SLC3A2* results in obvious oxidative damage and relates to melanocyte ferrocytosis. (**A**) Expression level of SLC3A2 was reduced, and SLC3A2 was downregulated in *SLC3A2*-interference group. Gel images were cropped for display. (**B**) Inhibition of melanocytes in G1 phase after *SLC3A2* interference. (**C**) *SLC3A2*-interference group showed significant decrease in cell proliferation rate and significant increase in apoptosis rate and ROS content. In the *SLC3A2*-interference group, contents of Fe^2+^, MDA, GSH, and GPX4 expression in melanocytes increased, and SOD activity and TYR and COX2 expression levels decreased. Note: *SLC3A2*-interference vs. control, **: *P* < 0.01, ***: *P* < 0.001; *SLC3A2*-interference vs. *SLC3A2*-interference NC, ##: *P* < 0.01, ###: *P* < 0.001

To explore the function of the downregulated RBP gene *SLC3A2* in melanocytes, we transfected human primary melanocytes with an *SLC3A2*-interfering plasmid. Western blot was selected for further validation of SLC3A2 after the establishment of the interference cell model in vitro. SLC3A2 was significantly downregulated in the *SLC3A2*-interference group, and its expression level was significantly reduced (*P* < 0.05) (Fig. 6A; Additional File 8 and 9). After *SLC3A2* interference, L-DOPA staining showed that the melanin content decreased (Fig. 6B), proliferative ability of melanocytes decreased, apoptotic ability increased, ROS content increased significantly, and cells were inhibited in the G1 phase (early stage of DNA synthesis) (Fig. 6C).

The ferrous iron (Fe^2+^) content, SOD activity, and MDA content in the cells of each group were determined using biochemical detection methods. Compared to the blank and *SLC3A2*-interference empty groups, *SLC3A2* interference decreased the SOD activity and increased the Fe^2+^ and MDA content in melanocytes (Fig. 6D). Compared with those in the blank and *SLC3A2*-interference empty groups, the expression levels of TYR and COX2 in the *SLC3A2*-interference group were significantly increased, whereas the expression levels of glutathione (GSH) and GPX4 showed the opposite trend (Fig. 6D). The above results confirm that knocking out *SLC3A2* results in obvious oxidative damage in melanocytes, and downregulating *SLC3A2* is related to melanocyte ferrocytosis.

## Discussion

The pathogenesis of vitiligo results from the joint action of skin cell groups of the whole thickness, such as keratinocytes, fibroblasts, and immune cells [6, 20]. In the present study, scRNA-seq data were used to identify cell subgroups and label key molecules in the skin tissues of patients with vitiligo and healthy people and to reveal key cell lineages in the skin of patients with vitiligo by looking for key cell subgroups and functional genes [21]. Overall, 28 cell subgroups were identified in 14 cell types, and the same cell types had different subgroups, suggesting certain differences in the functions of the same cell types in vitiligo lesion tissue.

This study found significant differences in the relative ratio of each cell subpopulation in the skin between patients with vitiligo and healthy individuals. Compared with that in the healthy group, the proportion of C8 (memory CD8 + T cells), C14/C20 (CD8 + NKT cells), C4 (memory CD4 + T cells), and C27 (mast cell) cell subpopulations increased significantly in patients with vitiligo, while the numbers of C26/C12 melanocytes and almost all keratinocyte subpopulations decreased significantly. Melanocytes were missing in vitiligo lesions. The single-cell sequencing samples were taken from the junction between damaged and normal skin and contain some melanocytes. This is the reason why the vitiligo group has melanocytes, but the number was decreased [10]. These results provide a panorama of the changes in cellular composition between vitiligo and normal human skin, indicating potential functional differences between the different cell types. Previous research has also revealed changes in the cell groups present in the skin of patients with vitiligo. For example, vitiligo is an autoimmune disease mediated by CD8 + T cells triggered by oxidative stress [22], and their number may correlate with the degree of disease [23]. Further recruitment of cytotoxic T lymphocytes produces more interferon-γ, directly inhibiting melanin production [24].

Keratinocytes synthesize and secrete various cytokines that directly or indirectly regulate melanocyte proliferation and melanin synthesis and transport after binding to specific receptors via paracrine action. Keratinocytes from patients with vitiligo are prone to apoptosis. The low expression of various cytokines leads to melanocyte death [25, 26]. In the unsupervised clustering analysis of RBP-expressing cell populations, we found that groups expressing cluster12 keratinocytes and cluster8 melanocytes were aggregated in the apoptosis pathway, suggesting the existence of common RBPs that induce the apoptosis of both keratinocytes and melanocytes. When treating vitiligo using melanocyte transplantation, some scholars have co-cultured melanocytes and keratinocytes to improve the success rate of transplantation [27]. Our findings will provide a reference for studying the specific underlying mechanisms.

RBPs regulate nearly every aspect of mRNA processing and are important regulators of cellular gene expression. RBPs are also involved in vitiligo pathogenesis [28]. The present study is the first to analyze the expression and regulatory patterns of RBPs in different cell types at the single-cell level in patients with vitiligo and healthy individuals. The expression patterns of RBPs were highly heterogeneous among different cell types and different cell populations could be distinguished based on RBP-expression patterns. In addition, we found that in some cell types, such as keratinocytes, Langerhans cells, and melanocytes, the dominant RBP-expression pattern was different between healthy individuals and patients with vitiligo. In the vitiligo group, the specifically increased melanocyte RBP-expression clusters were cluster8 and cluster21, and the marker RBPs of these clusters were enriched in the apoptosis- and immune-related pathways, respectively. Melanocyte apoptosis and abnormal immune regulation are important mechanisms involved in vitiligo [29]. The roles of RBPs in apoptosis- and immune-related pathways in vitiligo pathogenesis remain unclear and require further study.

Analyzing the RBPs differentially expressed in different cell types between the patients with vitiligo and healthy groups indicated more differences in RB expression, especially in melanocytes, CD8 + NKT cells, endothelial cells, B cells, and smooth muscle cells. Among them, some upregulated and downregulated RBP genes differed among cell types, such as *DCD, RPS26, RPS4Y1, HLA-A, S100A4, HSPA1A, HSP90AA1, JUN, LGALS3*, and *HSPD1*.

The frequency of *HLA-A2* is increased in patients with vitiligo [30]. The *HSPA1A* G/C and *HSPA1B* A/G variants were not associated with susceptibility to vitiligo. By contrast, *HSPA1L* T/C is associated with protection against vitiligo [31]. *HSP90AA1* affects important biological processes, such as melanogenesis in melanocytes and cellular oxidative stress [32]. Among the differentially expressed RBPs upregulated in the melanocyte C2 and C26 populations, some genes related to the cell cycle or immune inflammation, such as *S100A4* and *BST2*, were included. We extracted the top 5 upregulated RBP genes in the patients with vitiligo and healthy individuals in melanocytes and predicted their co-expressed genes; the target genes of *HLA-A*, *IFIT3*, *BST2*, and *WARS* were significantly enriched in the immunity, interferon-related, antigen presentation, and other pathways. *RPS4Y1* was enriched in the redox, cell proliferation, aging, and other pathways, whereas *S100A4* was enriched in the extracellular matrix, aging, platelet degranulation, and other pathways. These results indicated that the expression of various functional RBP genes in melanocytes is regulated during vitiligo development.

Using bulk RNA-seq data from melanocytes, we validated the RBPs that differed in melanocytes at the single-cell level between patients with vitiligo and healthy controls. Eight RBP genes were upregulated, including *CDKN2A*, *HLA-A*, *RPL12*, *RPL29*, *RPL31*, *RPS19*, *RPS21*, and *RPS28*, while one, *SLC3A2*, was downregulated. There was a large difference in the detection of RBP at the tissue and cell-line levels, which may be related to the dynamic regulation between cells and also shows that the cell-line level does not fully reflect the pathology and mechanism in the tissue. Among the upregulated RBP genes, *CDKN2A* is involved in regulating the melanocyte cycle [33], and *HLA-A2* gene frequency is increased in patients with vitiligo [30]. Other ribosomal protein genes have not been reported in vitiligo, and further research is needed.

SLC3A2 is an 85 kDa type-II transmembrane glycoprotein [34], which usually acts as a chaperone, forms a heterodimer with an amino acid transporter, such as SLC7A11, and functions in the cell membrane [35]. The cystine/glutamate transporter system, composed of SLC3A2 and SLC7A11, plays an important role in regulating ferroptosis; its inhibition restricts its uptake ability, thereby blocking the synthesis of GSH and decreasing the cellular antioxidant capacity and ferroptosis [36]. We further confirmed through cytological experiments that knocking down *SLC3A2* had an obvious oxidative damaging effect on melanocytes: the melanocyte ROS content increased significantly, SOD activity decreased, and MDA content increased. Following *SLC3A2* interference, the iron content of melanocytes and expression levels of TYR and COX2 increased considerably, and the expression levels of GSH and GPX4 decreased, suggesting that *SLC3A2* downregulation is involved in melanocyte ferroptosis [37]. Tumor immunotherapy studies have shown that interferons released from CD8 + T cells can downregulate *SLC3A2* and promote tumor cell ferroptosis [38]. However, whether *SLC3A2*-induced ferroptosis in melanocytes is related to the interferons released from CD8 + T cells remains unclear.

To the best of our knowledge, there have been few studies on RBPs in vitiligo and no analyses of RBPs at the single-cell level. In this study, we used bulk RNA-seq to verify that RBP genes are associated with vitiligo development in melanocytes and the function of SLC3A2 at the cellular level. Therefore, this is the first study to demonstrate a relationship between ferroptosis and *SLC3A2* expression in vitiligo melanocytes. These findings provide a new approach to studying the pathogenesis of vitiligo and a new means for targeted therapy. Despite these promising findings, this study had some limitations. First, we used only one dataset to screen for the differential genes. Second, few cell-function experiments were conducted. Finally, in vivo, experiments were not performed to verify this function. Future studies should explore the underlying mechanism between *SLC3A2* expression and the prognosis of vitiligo.

## Conclusions

This study is the first to analyze skin samples from patients with vitiligo and healthy individuals in terms of RBP expression and regulatory patterns in different cell types at the single-cell level. Melanocyte-related RBPs were identified, and it was confirmed that *SLC3A2* interference induced melanocyte ferroptosis, providing a new avenue for research on the pathogenesis of vitiligo.

## Materials and methods

### Retrieval and processing of public data

The unique molecular identifier (UMI) count matrix of the scRNA-seq data of skin samples from 5 healthy donors and 10 patients with vitiligo was downloaded from the CNCB database (PRJCA006797; https://ngdc.cncb.ac.cn/omix/release/OMIX691) [10]. Accessed 10 June 2022. The UMI count matrix was converted into a Seurat object using the R package Seurat (version 4.0.4) [39]. Cells with mitochondrial-derived UMI counts > 15%, detected genes < 500, or UMI numbers < 1000 were considered low-quality cells and removed. Genes detected in fewer than five cells were removed from downstream analyses.

### Preprocessing of scRNA-seq data

The UMI count matrix was log-normalized after quality control. The top 2000 variable genes were used to create potential anchors using the FindIntegrationAnchors function in Seurat. The integrateData function was used to integrate the data. Principal component analysis was performed on an integrated data matrix. The top 50 PCs were used for downstream analyses with the elbow plot function. The major cell clusters were identified using the FindClusters function (resolution = 0.4). The cells were classified into 17 major cell types. They were then visualized using tSNE or UMAP plots. To classify the cell type within each cluster, we employed the FindMarkers function to identify the gene markers specific to each cell cluster [10]. Additionally, we utilized the ScType tools to assign and annotate cell types accurately [40].

### Analysis of differentially expressed genes

DEGs were determined using the one-tailed Wilcoxon rank-sum test with the FindMarkers/FindAllMarkers function in the Seurat package. The Bonferroni correction was used to adjust *p*-values. Adjusted *p*-values < 0.05 indicated significance. To determine the DEGs, all genes were probed. The difference in the percentage of detected cells was at least 0.15, and the difference in expression on a natural log scale was at least 0.5.

### Cluster analysis of RBP genes

A catalog of 2,141 RBPs was retrieved from four previous reports [41–44]. The UMI count matrix of the RBPs was extracted as input for Seurat for the cell cluster. FindAllMarkers was used to select differentially activated RBPs. The co-expression association network of RBPs and targeted genes was constructed using the “grn” algorithm from the SCENIC python workflow (version 0.11.2) using default parameters [45]. The RBP target genes and their networks of modules were visualized using Cytoscape (v3.9.1) (https://cytoscape.org/). Accessed 21 June 2022.

### Retrieval and processing of bulk RNA-seq data

Public sequence data files for the bulk RNA-seq of melanocyte cell lines were downloaded from the SRA (https://www.ncbi.nlm.nih.gov/bioproject/PRJNA733523) [17]. Accessed 21 June 2022. The NCBI SRA Tool fastq-dump was used to convert the SRA Run files to fastq format. The raw reads were trimmed using the FASTX-Toolkit (v.0.0.13, http://hannonlab.cshl.edu/fastx_toolkit/). The clean reads were evaluated using FastQC (http://www.bioinformatics.babraham.ac.uk/projects/fastqc). Accessed 21 June 2022.

### Read alignment and differentially expressed gene analysis

The clean reads were aligned to the human genome using HISAT2 [46]. The uniquely mapped reads were used to calculate the read number of each gene and the fragments per kilobase of exon per million fragments mapped, which was used to evaluate the expression levels of these genes. DEseq2 software was applied to screen the raw count data for DEGs [47]. To determine whether a gene was differentially expressed, the results were analyzed based on a false discovery rate ≤ 0.05 and fold change ≥ 2 or ≤ 0.5. Using a catalog of 2,141 RBPs, the expression profiles of differentially expressed RBPs were filtered from those of all DEGs.

### Functional enrichment analysis

Gene Ontology terms and Kyoto Encyclopedia of Genes and Genomes pathways were used to identify the functional categories of the genes using KOBAS 2.0 [48]. The Benjamini–Hochberg false discovery rate-controlling procedure and hypergeometric test were used to define the enrichment of each term.

### Cell culture and small interfering RNA transfection

Foreskin specimens of young males in the urology outpatient department were collected, rinsed with phosphate-buffered saline (PBS) containing streptomycin and penicillin, and transferred to sterile Petri dishes. PBS was added, and the dermis and subcutaneous fat tissue were removed and rinsed with PBS. The washed specimens were cut into pieces, followed by adding 1 mL of 0.25% trypsin. The specimens were then placed in a refrigerator at 4 °C for 12–16 h. After the digestion was terminated, a cell suspension was prepared and filtered through a cell sieve. The cell suspension was collected and centrifuged at 1500 × *g* for 2 min, and the cells were inoculated into a sterile culture bottle, followed by adding 8 ml melanocyte culture medium (Promocell, Heidelberg, Germany).

The cells were cultured at a constant temperature of 37 °C and 5% CO_2_. The medium was changed for the first time after 2 d, then after each day. The cells were observed under an inverted microscope (SOPTOP, Ningbo, China). When 80% of the area of the bottom of the culture bottle was covered with melanocytes, the cells were subcultured. After washing the melanocytes, trypsin was added, and the cells were observed under the inverted microscope. When the dendrites of the melanocytes retracted, the cell bodies became round, and most of the cells were suspended. The cell suspension was collected and centrifuged at 1500 × *g* for 2 min with a centrifugal radius of 10 cm, and the supernatant was discarded. M2 melanocyte culture medium was added, inoculated into two new sterile culture bottles, and cultured in a constant-temperature incubator (Crystal Technology & Industries, Inc, Addison, TX, USA) at 37 °C in 5% CO_2_.

Empty SLC3A2-small interfering RNA (siRNA) and SLC3A2 interference plasmids were constructed (Beijing Tsingke Biotech Co., Ltd., China). The cells were plated in a 6-well plate the day before the transfection experiment started. Transfection was initiated when the cell lines reached a confluency of 70–80%. The experimental procedure was as follows: (1) The serum-free medium was changed 2 h before the transfection experiment started. (2) The transfection solution was prepared. The following two solutions were prepared in EP tubes (the amount used to transfect cells in each well): Solution A—30 pmol/mL siRNA was diluted with 150 µL of fetal bovine serum-free medium. A pipette gun was used for mixing. The mixture was incubated for 5 min at 25 ℃ on a clean bench. Solution B—Lipofectamine 2000 (Invitrogen, Carlsbad, CA, USA) was shaken gently before use; 5 µL Lipofectamine 2000 was mixed in 150 µL fetal bovine serum-free medium using a pipette gun, and the mixture was incubated at 25 ℃ for 5 min. (3) Solution A was added to Solution B, flicked to ensure proper mixing, and placed at 24–26℃ for 20 min. (4) The cells were taken out of the incubator, and the mixture of A and B was added slowly into the culture medium. The resulting mixture was shaken well and placed in the incubator at 37 ℃ for 6 h. The culture was allowed to grow further for 48 h. (5) Protein analysis and functional experiments were performed using digested cells 48 h after seeding. The SLC3A2-siRNA sequence was as follows: 5′-CGGTTGCTGGTGCCGTGGTCATAACTCGAGTTATGACCACGGCACCAGCAATTTTTT-3′, and the carrier was pLKO.1-copGFP-PURO.

### Quantitative reverse-transcription polymerase chain reaction

The total RNA was extracted from the melanocytes using the TRIzol method and quantified using a NanoDrop spectrophotometer (NanoDrop Technologies, Wilmington, DE, USA). cDNA was synthesized from total RNA using the Evo M-MLV RT master mix (Accurate Biology, Changsha, China). Then, quantitative reverse-transcription polymerase chain reaction (qRT–PCR) was performed on a QuantStudio Real-Time PCR System (Thermo Fisher Scientific, Waltham, MA, USA) using SYBR Green Pro Taq HS qPCR Kit II and ROX dye (Accurate Biology). The relative gene expression levels were normalized to GAPDH, and fluorescence quantification was performed using the 2^–∆∆Ct^ method. Three replicates were used for each sample. The primer sequences used in our study were as follows: actin (human)-F-CCCATCTATGAGGGTTACGC, actin (human)-R-TTTAATGTCACGCACGATTTC, SLC3A2-F-TGAATGAGTTAGAGCCCGAGA, and SLC3A2-R-GTCTTCCGCCACCTTGATCTT.

### Western blotting

EDTA trypsin was used to digest the cells, and RIPA lysis buffer (R0010; Solarbio, Shanghai, China) was used to lyse the melanocytes to obtain proteins. The protein was quantified using the BCA method, 12% SDS polyacrylamide gel electrophoresis (Biosharp, China), 5% BSA blocking (Biofroxx, Germany), and primary antibody SLC3A2 (ABclonal, USA) overnight at 4 °C. The secondary antibody Rb a beta-actin (Bioss, China) was incubated on a horizontal decolorizing shaker at room temperature for 1 h. The protein band pattern was identified using a chemiluminescence system (Beijing Liuyi Biotechnology Co., Ltd., China).

### Detection of cell function

After the plasmid was successfully transfected, the cells were seeded in a 96-well plate and cultured for 24 h. A CCK-8 assay (Beijing 4 A Biotech Co., Ltd, China) was performed. Five replicates were set up, and the absorbance at 450 nm was measured using a microplate reader (Beijing PERLONG Co., Ltd, China). The cell apoptosis rate, cell cycle stage, and ROS production level (Solarbio) were detected through flow cytometry, and three replicates were used.

### Detection of ferroptosis-related indicators

Total SOD activity and lipid oxidation detection kits (Beyotime, Shanghai, China) were used to detect the levels of SOD and MDA, which are related to oxidative stress. L-DOPA staining was used to detect the melanin content (KALANG, Shanghai, China). Corresponding ELISA kits (Jianglaibio, Shanghai) were used to detect the levels of cellular tyrosine protein (TYR), GSH, COX2, and GPX4, and an iron content detection kit (Mlbio, Shanghai, China) was used to detect the cellular iron ion content. The experimental procedures were performed according to the manufacturer’s instructions, and each experiment was repeated three times.

### Statistical analysis

The pheatmap package (https://cran.r-project.org/web/packages/pheatmap/index.html) in R was used to perform clustering based on Euclidean distance. Student’s t-test was used for comparisons between the two groups. The western blot result strip processing involved analyzing the grayscale values of each strip from the original image using ImageJ 1.0. The grayscale values of the target band/internal reference protein were used for analysis, whereas the rest were the actual measurement data. The data are represented as (x ± SD) and were entered into GraphPad Prism 7.00 software for processing (GraphPad Inc., La Jolla, CA, USA). Single-factor analysis of variance (F-test) was used to compare multiple groups (ɑ = 0.05, with *P* < 0.05 indicated significant differences).

### Electronic supplementary material

Below is the link to the electronic supplementary material.

Supplementary Material 1

Supplementary Material 2

Supplementary Material 3

Supplementary Material 4

Supplementary Material 5

Supplementary Material 6

Supplementary Material 7

Supplementary Material 8

Supplementary Material 9

## Data Availability

All data generated or analyzed during this study are included in this article and its supplemental information files, and are available from the corresponding author on reasonable request.

## Abbreviations

RBPs: RNA-binding proteins
RNA-seq: RNA sequencing
scRNA-seq: Single-cell transcriptome sequencing
siRNA: Small interfering RNA
DEGs: Differentially expressed genes
qRT–PCR: Small interfering RNAQuantitative reverse transcription polymerase chain reaction
SRA: Sequence Read Archive
UMI: Unique molecular identifier
PC: Principal component
GSH: Glutathione
PBS: Phosphate-buffered saline
