# Supplementary material for: Identification and validation of RNA-binding protein SLC3A2 regulates melanocyte ferroptosis in vitiligo by integrated analysis of single-cell and bulk RNA-sequencing
Source: BMC Genomics. 2024 Mar 4;25:236. doi: 10.1186/s12864-024-10147-y (PMC10910712; doi:10.1186/s12864-024-10147-y)
Supplement: Supplementary file 4 — Supplementary Material 4 [file 12864_2024_10147_MOESM4_ESM.docx]

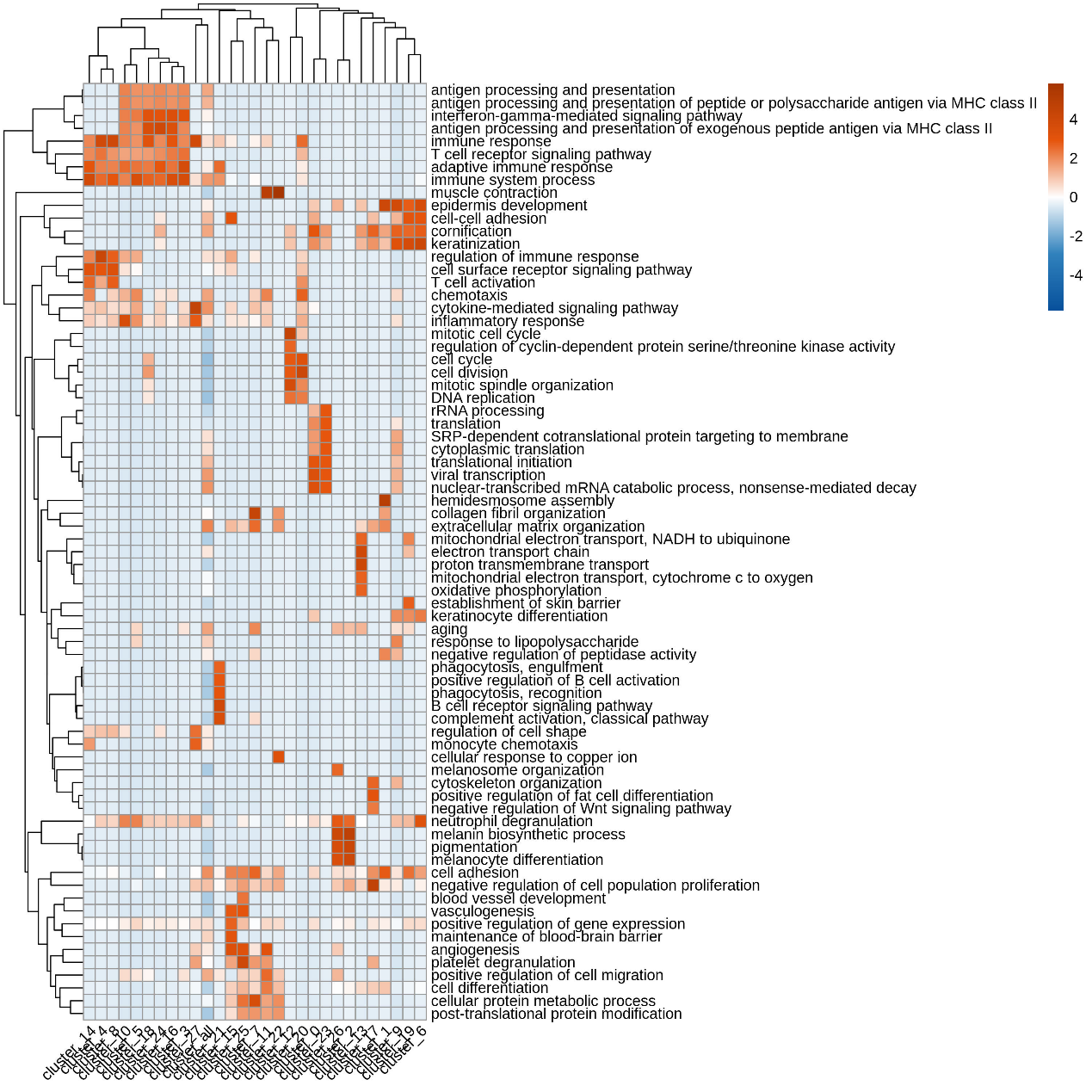


**Additional file 4** Gene Ontology enrichment analysis of biological processes of top 100 marker genes of each cell type. Top three terms were selected for each cluster, and heatmap shows enrichment q-value of these terms (scaled by column).
