# Supplementary figures and images for: Identification and validation of RNA-binding protein SLC3A2 regulates melanocyte ferroptosis in vitiligo by integrated analysis of single-cell and bulk RNA-sequencing
Source: BMC Genomics. 2024 Mar 4;25:236. doi: 10.1186/s12864-024-10147-y (PMC10910712; doi:10.1186/s12864-024-10147-y)

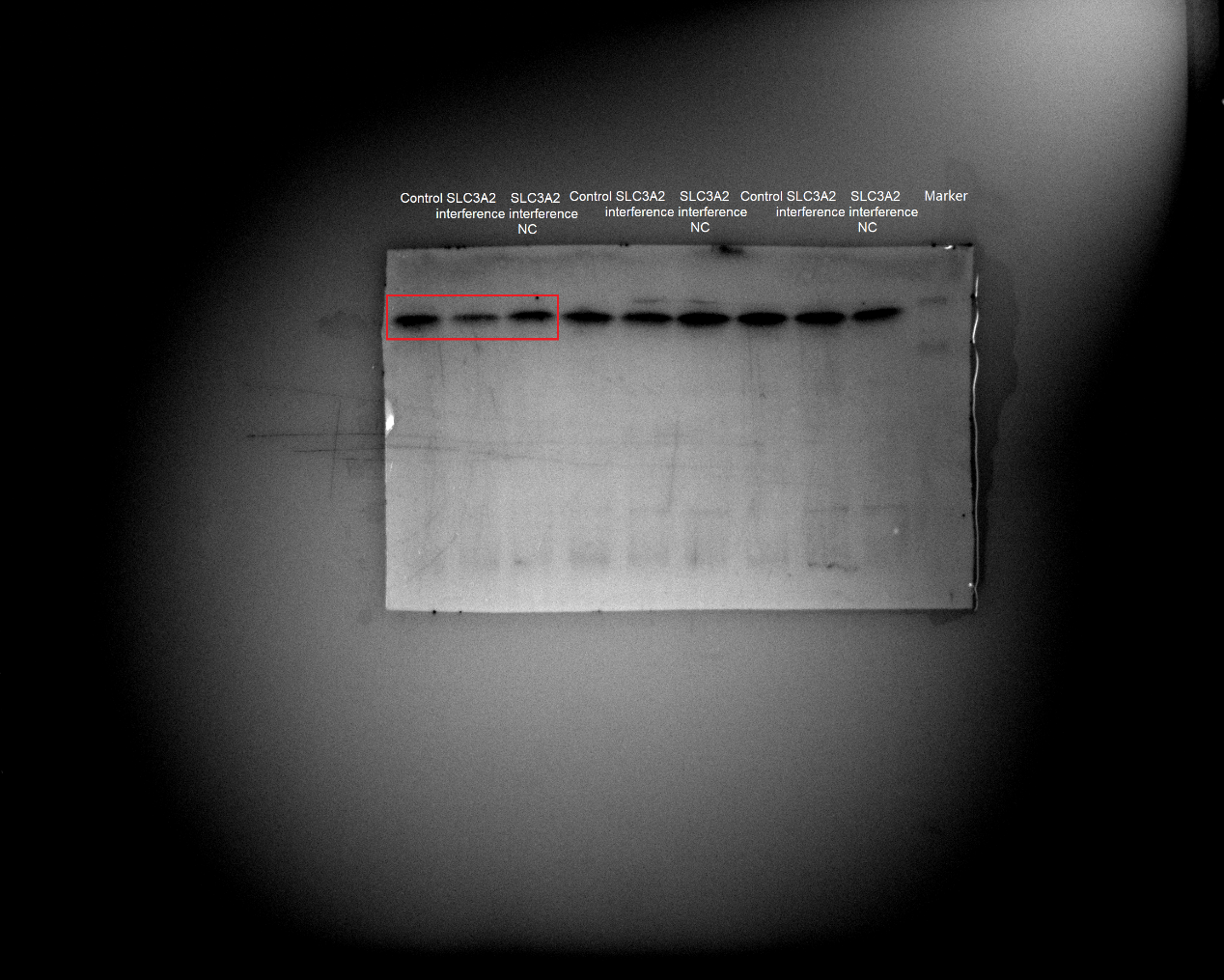


**Additional file 8** Original, unprocessed versions for Western blot of SLC3A2.

Supplement: Supplementary file 8 — Supplementary Material 8 [file 12864_2024_10147_MOESM8_ESM.docx]

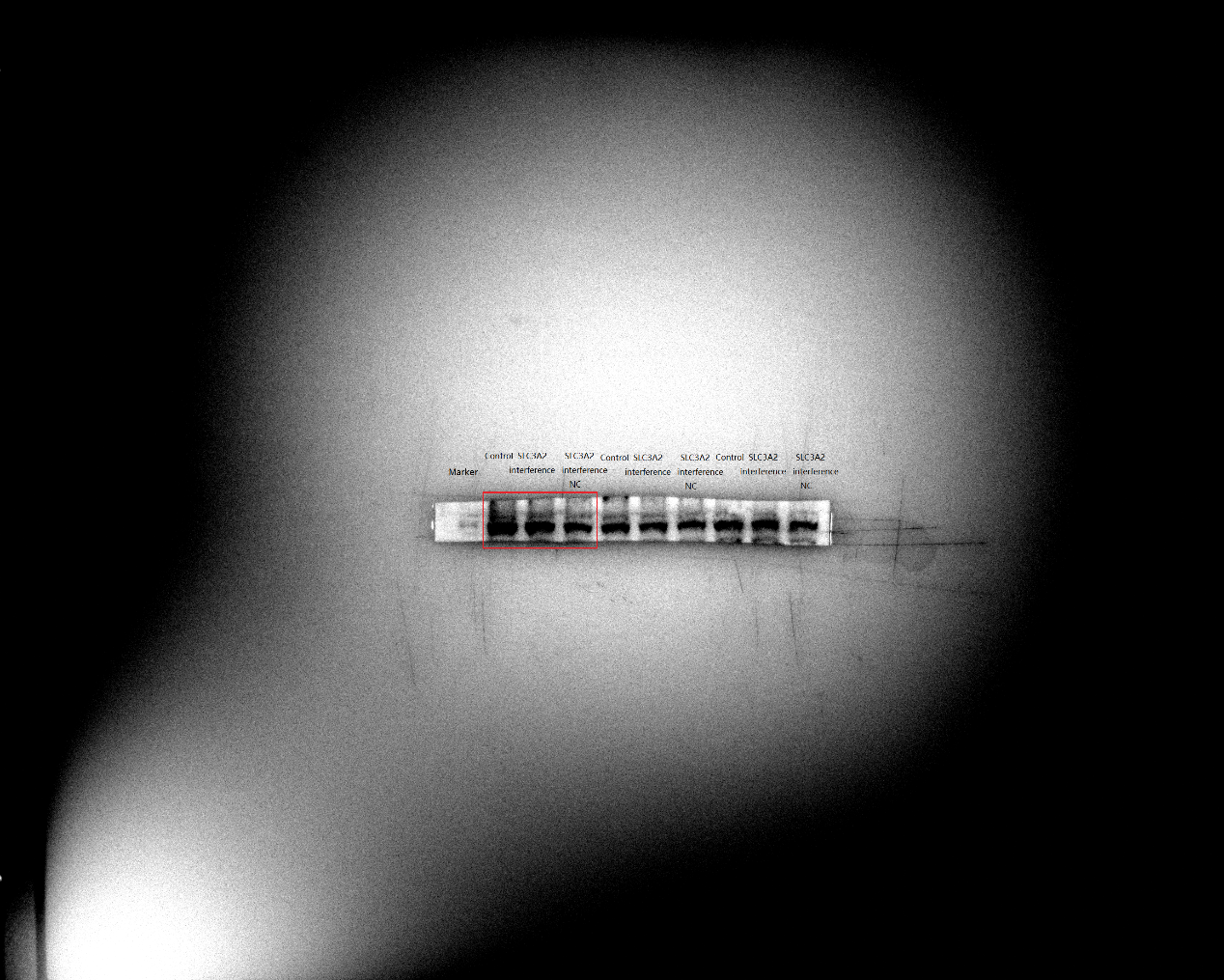


**Additional file 9** Original, unprocessed versions for Western blot of β-actin.

Supplement: Supplementary file 9 — Supplementary Material 9 [file 12864_2024_10147_MOESM9_ESM.docx]
